# Dynamic Locking Plate versus Multiple Cancellous Screws for the Fixation of Intracapsular Femoral Neck Fractures: Long-Term Results and Quality-Of-Life Assessment Based on Patient-Reported Outcome Measures

**DOI:** 10.3390/jcm13041123

**Published:** 2024-02-16

**Authors:** Yoav Krupik, Sagie Haziza, Ran Thein

**Affiliations:** 1 Department of Orthopedic Surgery, Chaim Sheba Medical Center, Tel Hashomer and Sackler Faculty of Medicine, Tel Aviv University, Tel Aviv 5265601, Israel; 2 Department of Orthopedic Surgery, Rutgers New Jersey Medical School, University Hospital, Newark, NJ 07103, USA; sagiehaziza@gmail.com

**Keywords:** hip fracture, internal fixation, unstable intracapsular fracture, PROMs

## Abstract

The purpose of this study was to compare the long-term clinical outcomes and quality-of-life measures for two fixation methods in the setting of displaced femoral neck fractures. The two groups included fixation with multiple cancellous screws (group 1) and telescopic femoral neck screws and a small locking plate device (Targon FN) (group 2). Patients underwent reduction and internal fixation with either multiple cancellous screws or the Targon FN device from March 2000 to January 2012. Failure endpoints included nonunion, osteonecrosis of the femoral head, and revision surgery. Patient-reported outcome measures included chronic pain, ability to ambulate, and the use of ambulation assistive devices. Statistical analysis demonstrated a statistically significant lower rate of non-union and overall complication in the Targon FN group (*p* value < 0.001 and *p* value = 0.005, respectively). Logistic regression analysis showed that operative fixation with the Targon FN device decreased the odds ratio for overall complication by a factor of 0.34 (*p* = 0.02). There were no statistically significant differences between groups 1 and 2 in patient-reported outcomes (chronic pain (*p* = 0.21), ability to ambulate (*p* = 0.07), and the use of an ambulation assistive device (*p* = 0.07)). When compared to traditional cancellous screw fixation of femoral neck fractures, the Targon FN device has significantly lower complication rates and equivalent patient-reported outcomes.

## 1. Introduction

Displaced intracapsular femoral neck fractures in young patients pose a unique challenge when compared to similar injury in the elderly; young patients are generally more active, have fewer medical problems, and have good bone quality. The goals of treatment in the young population focus on preserving the femoral head, avoiding osteonecrosis, and achieving union [1].

For decades, clinical trials have explored the proper treatment of femoral neck fractures. Generally, the literature suggests young patients should undergo operative fixation. Conversely, surgeons should have a lower threshold for arthroplasty in the elderly. However, there is nuance when stratifying patients into treatment groups. Operative fixation requires less time under anesthesia and is associated with lower levels of blood loss compared to arthroplasty. Therefore, a surgeon may elect to complete operative fixation in their higher-risk patient with femoral neck fracture. The same is true for younger patients with poor bone stock or severely displaced fractures who are deemed to be better arthroplasty candidates. Furthermore, there is no consensus on which fixation method is most effective in the fixation group. Typically, fixation is completed using cannulated screws or sliding hip screw implants. Evidence suggests both methods are comparable, though there is conflicting research regarding non-union rates, patient satisfaction, operative length, and anesthesia time [1,2,3,4,5,6,7,8]. In recent years, alternative fixed angle implants have been introduced and have gained popularity [9,10].

One prominent fixed angle device is the Targon FN (B-Braun AG, Melsungen, Germany): a contoured locking titanium sideplate which can accommodate four proximal sliding screws, in addition to two distal locking screws. Since its emergence in 2007, studies, including two meta-analyses, have succeeded in showing good short-term outcomes for the device [11,12,13,14,15,16].

Although exhibiting impressive outcomes in the short term, to the best of our knowledge, there are no data regarding the Targon FN system’s complication rates through mid- or long-term follow-up. Moreover, there is a little research on long-term patient-reported outcome measures, including chronic pain, level of ambulation, and the use of ambulation assistive devices. These data are critical in evaluating the success of surgical treatment in young patients with displaced femoral neck fractures.

Therefore, we designed a retrospective review to evaluate mid- and long-term surgical outcomes of young patients with displaced femoral neck fractures fixed with the Targon FN as compared to fixation with multiple cancellous screws.

## 2. Patients and Methods

### 2.1. Patient Selection, Data Collection, Inclusion, and Exclusion Criteria

Between 2000 and 2012, 97 patients with unstable intracapsular femoral neck fractures (OTA classification 31-B2.3 or 31-B3.11) were hospitalized in the orthopedic department of Sheba medical center. Data on these patients were obtained via electronic medical records and related database review for study analysis. Fracture classifications and complication diagnosis were independently performed by two reviewers, with discrepancies resolved by a third orthopedic surgeon, all blinded to the surgery type. Exclusion criteria included polytrauma, pathological fractures, prior hip, or femur surgery on the operative side. The control group (group 1) comprised 47 patients treated with three cancellous screws fixation, and the study group (group 2) included 50 patients treated with the Targon FN device. Data were collected between March 2000 to January 2012, and follow-up was completed between July and October 2020.

### 2.2. Surgical Technique and Postoperative Protocol

**Figure 1:**
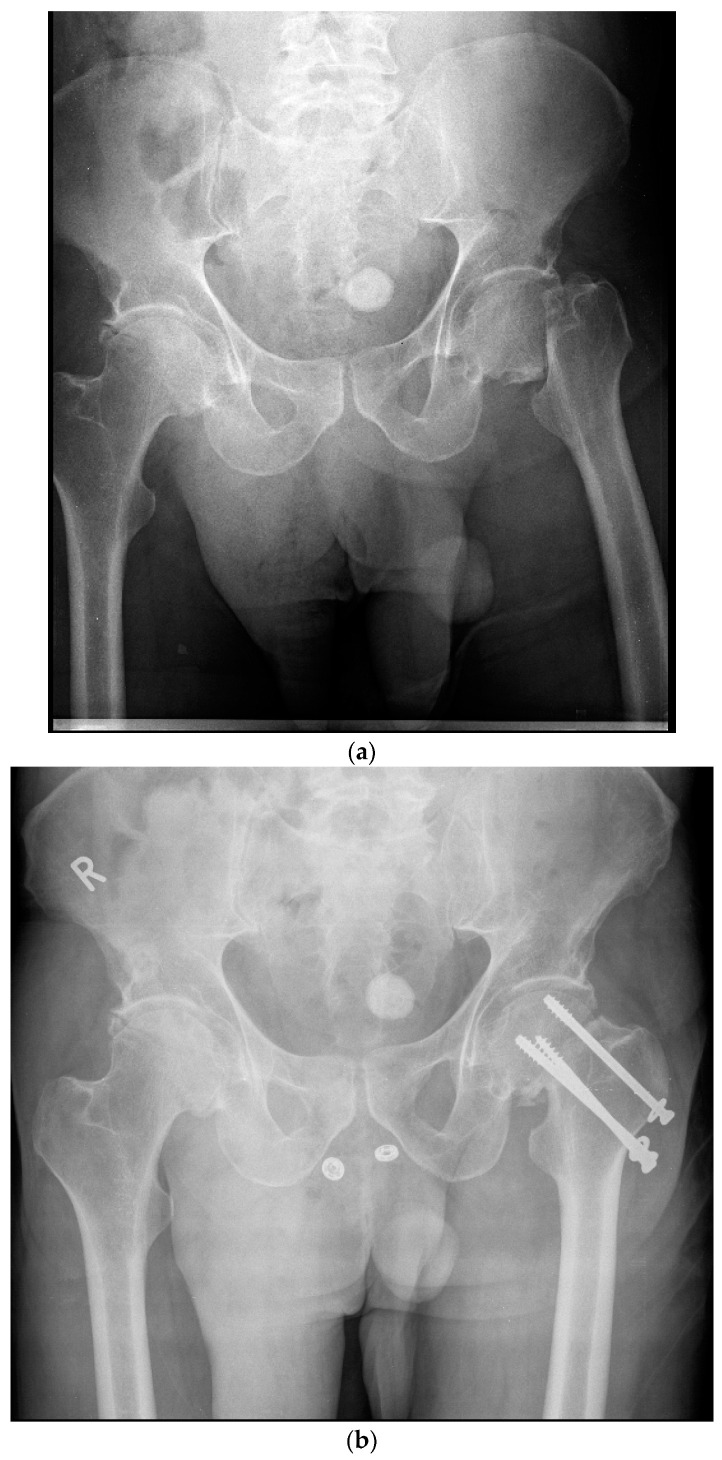
(**a**)—Pre-operative X-ray demonstrating a left sided displaced femoral neck in a 59-year-old male. (**b**)—Postoperative X-ray demonstrating operative fixation of the same fracture with cancellous screws. R—Patient’s right side.

**Figure 2:**
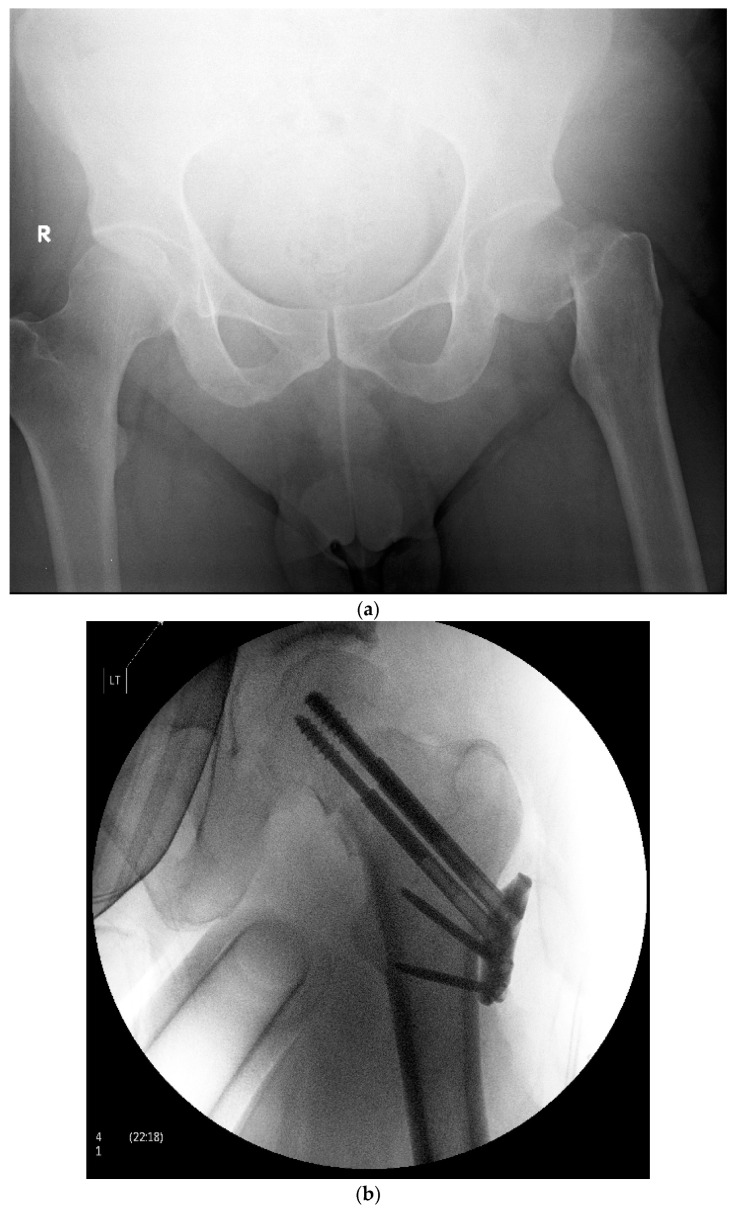
(**a**)—Pre-operative X-ray demonstrating a left sided displaced femoral neck fracture in a 48-year-old male. R—Patient’s right side. (**b**)—Postoperative X-ray demonstrating operative fixation of the same fracture with the Targon FN system. LT—Patient’s left side.

The mechanism of injury varied and included falls from height, motor vehicle collisions, and blunt trauma. The surgical technique has been previously described by Thein et al. [13] and Parker and Stedtfeld [4]. In summary, the patients in both groups were given either general or regional anesthesia and positioned supine on a fracture table. Using fluoroscopic aid, limb length was restored using gentle longitudinal traction. In most cases, correction of rotational deformity and proper cortical alignment were achieved with internal rotation and adduction of the effected lower limb. When necessary, an open approach to the femoral neck was utilized to achieve reduction. Acceptable reduction was achieved when the fracture was in anatomic reduction or slight valgus on the anterior–posterior view with no extension or flexion on the axial view. For the multiple cancellous screw group, a guide wire was inserted just above the level of the lesser trochanter. Three screws were then inserted up to the subchondral bone of the femoral head following guide wires in an inverted triangle or another triangular shape by the surgeon (Figure 1). For the Targon-FN-treated group, the reduction target was slight valgus or anatomic reduction on the anterior–posterior view and no extension or flexion on the axial. The femur was approached directly via a 4–6 cm lateral incision. Guide wires and telescopic screws were then sequentially inserted to attach the sideplate to the femur to achieve proximal and distal fixation (Figure 2). The majority of surgeries were performed by 3 surgeons, all of them senior consultants with more than 10 years of experience. They were all members of the same department and worked according to the same standards. Restricted weight bearing was encouraged where possible for all patients. Full weight bearing was allowed after reassessment 6 weeks after surgery.

### 2.3. Follow-Up and Endpoints

Patient follow-up was completed using telephone calls during July–October 2020. Data regarding patients who could not be reached by telephone were obtained using their most recent outpatient clinical record. The patients’ quality-of-life measures were assessed using standardized EQ-5D-5L PROMs (Patient-Reported Outcome Measures) questionnaires. The EQ-5D-5L PROMs questionnaire was chosen because of its comprehensive coverage of health dimensions that allows for a holistic evaluation of an individual’s health status, its sensitivity to changes in health over time, simplicity in administration, and the fact that it captures the patient’s own perspective on their health, enhancing the relevance and applicability of the instrument across diverse populations and health conditions. In addition, they were asked about any follow-up surgery, revision surgery, or postoperative complication (osteonecrosis, nonunion, limb shortening) during the period between their initial surgery and present time. Complication endpoints included nonunion, osteonecrosis of the femoral head, and salvage (osteotomy or removal of internal fixation) or reconstruction (hemiarthroplasty or total hip replacement) revision.

### 2.4. IRB Approval

Approval for the study was obtained from our local Institutional Review Board. Screened patients received an early notice via mail and expressed an informed consent via telephone to participate in this study.

### 2.5. Statistical Analysis

Data were analyzed using RStudio software (RStudio, Inc., Boston, MA, USA). Categorical variables are given as number and percent. Continuous variables are given as mean and standard deviation (SD). The χ^2^ was used to test for statistical significance among categorical variables. The Brunner–Munzel test was used to test for statistical significance among ordinal variables, including chronic pain, ability to ambulate, and ambulation assistive device usage scores. Total major complications were calculated as the sum of non-union, avascular necrosis (AVN), periprosthetic fractures, or shortening of the effected limb. Revision surgery rates were separately assessed. Logistic regression was used to estimate the influence of the internal fixation device (independent variable) on major complication probability (dependent variable). All *p* values were 2-sided, and a *p* value of <0.05 was considered statistically significant.

## 3. Results

The study included a total of 97 patients, and their relevant demographic, clinical, and functional data are presented in Table 1. The mean age of the patients was 55.2 years (SD: 10.49, interquartile range 50–72). A total of 83% of patients were <65 years of age. The rest of the patients were offered operative fixation opposed to arthroplasty because of their clinical and radiographic presentation. Those >65 were active, physiologically young patients with high-quality bone. The mean duration of follow-up was 79.6 months for group 1 and 82.75 for group 2 (*p* value = 0.97).

Patients’ complication rates are presented in Table 2. Three patients from group 1 (6.38%) and seven patients from group 2 (14%) developed osteonecrosis of the femoral head (*p* value = 0.36). Seventeen patients (36.17%) had nonunion in group 1, while only two (4%) had nonunion in group 2 (*p* value < 0.001). Thirteen group 1 patients (27.65%) required revision surgery in comparison to only nine group 2 patients (*p* value = 0.25). Regarding only conversions to THR (subtracting hardware removals from total revision surgery count), group 1 and 2 had 13 and 8 cases, respectively (*p* value = 0.16).

When comparing total complication rate, including non-union, AVN, periprosthetic fractures, or shortening of the effected limb, group 1 had 23 complications (48.93%), while group 2 had 11 (22%) (*p* value = 0.005).

The logistic regression model found that performing internal fixation with the Targon FN device decreased the odds ratio for overall complication by a factor of 0.34 (*p* = 0.02).

Patients’ quality-of-life scores are exhibited in Table 3. In group 1, three patients reported no chronic pain since surgery, nine patients had mild pain, two had moderate pain, one had severe pain, and none had extreme pain (mean = 2.06). In the Targon FN treatment group, 7 patients reported no chronic pain, 16 mild pain, 7 moderate pain, 5 severe pain, and 3 extreme pain (mean = 2.5) (*p* value = 0.21).

Regarding ability to ambulate, seven group 1 patients reported no problem walking, four patients reported a slight problem walking, two a moderate problem, and one the inability to walk (mean = 1.85). In group 2, 30 patients reported no problem walking, 5 reported a slight problem, 1 a moderate problem, 1 a severe problem, and 1 reported the inability to walk (mean = 1.36) (*p* value = 0.07). When asked about the use of walking aids, seven group 1 patients reported no use of aids, three reported using one walking aid (cane), one reported using two aids, one reported on using a walker, and two reported the use of a wheelchair (mean = 2.14). In group 2, 29 patients reported no walking aids, 8 reported one walking aid, and 1 reported the use of a wheelchair (mean = 1.31) (*p* value = 0.07).

## 4. Discussion

The goal of treatment in patients with a displaced femoral neck fracture, especially young active individuals, is to preserve the femoral head and restore natural hip function. It has been reported that fracture healing with no osteonecrosis leads to a good functional outcome [17]. Furthermore, a more anatomic reduction leads to higher union and lower failure rates. In this retrospective study, we have presented long-term outcomes of femoral neck fracture fixation using the Targon FN by exploring the device’s failure rates and patients’ ability to return to pre-injury quality of life.

In summary, our study comprised 97 patients, predominantly under the age of 65, who underwent operative fixation for intracapsular femoral neck fractures. The two groups, categorized by the type of fixation method employed (group 1: conventional fixation, group 2: Targon FN device), were well-matched in terms of demographic and clinical characteristics. The mean follow-up duration was approximately 80 months in both groups. Group 2 demonstrated favorable outcomes with significantly lower nonunion rates and a reduced overall complication rate compared to group 1. Logistic regression analysis reinforced these findings, indicating a decreased odds ratio for overall complications in the Targon FN treatment group. Quality-of-life assessments revealed comparable pain levels between the groups, but group 2 exhibited slightly better ambulation ability and a trend towards lower use of walking aids. These results underscore the potential benefits of utilizing the Targon FN device in the operative fixation of intracapsular femoral neck fractures, emphasizing improved clinical outcomes and a reduced risk of complications.

The short-term failure rate of the Targon FN when used for internal fixation of displaced intracapsular femoral neck fractures ranges from 15 to 48% [16,18,19]. The literature has shown favorable results with the Targon FN compared to other methods of intracapsular femoral neck fracture fixation. Thein et al. have previously shown significant reductions in non-union rates, revision surgery, and overall complication rates in the short term with the Targon FN [13].

Biber et al. have concluded that using the Targon FN results in relatively low complication rates of 16.4% and a conversion to joint replacement of 9.6% in a cohort of 135 cases (mean age: 71 years; average operation time: 60 min; average hospital stay: ten days). Nevertheless, they concluded that the complication rate was significantly higher in displaced fractures and that implant perforation seems to be underestimated [20].

Though some researchers have explored the Targon FN success as compared to a control group in the past, the longest follow-up we could find is by Thein et al. of 28.6 months. Furthermore, to the best of our knowledge, no other paper has followed its patients for more than 10 years as in the current study. The longest follow-up we could find was in a project conducted by Majernícek M et al. in 2009, with a mean follow-up time of 6.9 years, examining the dynamic hip screw device’s outcomes, though the group was not compared to a control [7]. Both works also did not explore the impact of the Targon FN system on PROMs.

Our study results have strengthened past evidence regarding the Targon FN’s performance superiority in comparison with multiple cancellous screws. We have demonstrated that the Targon FN has significantly lower rates of total major complications (*p* value = 0.005). The greatest difference was seen specifically in non-union rates (*p* value < 0.001). However, the AVN incidence between the groups was not significantly different (*p* value = 0.37). The superiority of the Targon FN in nonunion rates is likely due to its effect on hip mechanics, while AVN is a pathology related to blood supply disruption. The Targon FN allows for a controlled collapse at the fracture site, while cancellous screws do not. For this reason, non-union complications are more common in the cancellous screw group in the shorter term. AVN was exposed at a later time frame secondary to the natural progression of poor blood supply in this fracture pattern. The long-term follow-up may also have influenced the non-significant difference in revision surgery and total hip replacement rates (*p* value = 0.25 and *p* value = 0.16, respectively). The hip replacement surgeries were at times conducted many years after the initial injury, more likely related to osteoarthritic changes and not to the original insult.

Concerning functional evaluation, studies have shown mixed results. Eschler et al. used the Harris Hip score to evaluate the Targon FN’s results compared to the sliding hip screw. They demonstrated a less favorable outcome (69.5 ± 14.5 points and 87.7 ± 13.9 points, respectively) [16]. Takigawa et al. have shown that 88.5% of the patients treated with the implant for intracapsular fractures had achieved their pre-injury level of mobility over an average 16.4-month follow-up [18]. Parker et al. reported that patients could maintain the same preoperative mobility score or achieve better mobility after femoral neck fracture fixation with the Targon FN [21,22].

To the best of our knowledge, this study was the first to report long-term quality-of-life measures in patients who underwent internal fixation of femoral neck fractures with the Targon FN as compared to internal fixation with multiple cancellous screws. Our data show increased ambulation ability and decreased use of ambulation assistive devices in the Targon FN group, though these results were not statistically significant (*p* value = 0.07 for both). There was no difference in chronic pain scores between the groups (*p* value = 0.21). These outcomes could be explained by the fact that we were more successful in contacting a larger group of Targon FN patients by telephone (30) compared to cancellous screw patients (14), while the rest of the data were obtained from follow-up clinical records as mentioned. Therefore, by the time of questioning, patients had aged significantly and likely suffered additional insults. Moreover, there is likely an element of healthy participant bias. Considering the previously discussed results, which exhibit the Targon FN’s superior performance over cancellous screws, we can assume that the patients that we were able to contact had an overall better natural history and were overall healthier than those we were unable to contact, which may bias our results in favor of the cancellous screws. Given these implications, the Targon FN trend towards improved PROMs in ambulation ability and reduced walking aid use is even more impressive.

Our study is not without limitations. Firstly, three different surgeons performed the operations and not a single surgeon (even though they were all senior consultants with more than 10 years of experience). It is a retrospective study and not a randomized control trial. We have mentioned the possibility of healthy participant bias above. We also have an uneven number of patients between the control and study group and specifically patients who we were able to contact using telephone calls, which may have influenced our analysis. Lastly, a logistics regression analysis is less significant in this small of a sample size. However, the analysis was meant to underscore the finding of lower total complication rate in Targon FN fixation as compared to cancellous screw fixation for femoral neck fractures.

We conclude that a displaced intracapsular femoral neck fracture, especially in young active patients, is a major traumatic insult and may represent a life-changing event. Older methods of fixation were associated with a relatively high rate of failure. Our paper demonstrates that the Targon FN has fewer major post-surgical complication rates compared to traditional fixation methods in the long term. Regarding quality-of-life measures, while not statistically significant, the Targon FN exhibits a positive trend towards the ability to preserve stable and satisfactory walking ability.

## Figures and Tables

**Table 1:** Demographic, clinical, and functional characteristics of the 97 patients in the two treatment groups. The χ^2^ was used to test for statistical significance among categorical variables, whereas the Brunner–Munzel test was used to test for statistical significance among ordinal variables. All *p* values were two-sided, and a *p* value of <0.05 was considered statistically significant. FN—femoral nail.

	Pinning Procedure (*n* = 47)	Targon FN (*n* = 50)	*p* Value
Characteristic			
Gender—*n* (%)			
Male	35 (74.46%)	24 (48%)	0.007
Female	12 (25.53%)	26 (52%)	
Pre-surgery walking ability—no walking aids—*n* (%)	44 (93.61%)	49 (98%)	0.27
Garden 4 fractures out of total number of displaced fractures—*n* (%)	30 (63.82%)	24 (48%)	0.11
Age at surgery—mean	56.3	54.2	0.63
Age group at surgery—*n* (%)			
Age < 50	9 (19.14%)	14 (28%)	0.3
Age ≥ 50	38 (80.85%)	36 (72%)	
Follow-up duration (months)—mean	79.6	82.75	0.96

Categorial variables are presented as count (percent). Continuous variables are presented as mean.

**Table 2:** Comparing postoperative outcomes and complications between the two treatment groups. The χ^2^ was used to test for statistical significance among categorical variables. All *p* values were two-sided, and a *p* value of <0.05 was considered statistically significant. FN—femoral nail, THR—total hip replacement.

	Pinning Procedure (*n* = 47)	Targon FN (*n* = 50)	*p* Value
Outcomes—*n* (%)			
Avascular necrosis	3 (6.38%)	7 (14%)	0.36
Nonunion	17 (36.17%)	2 (4%)	<0.001
Periprosthetic fracture	1 (2.12%)	1 (2%)	0.96
Limb shortening	2 (4.25%)	1 (2%)	0.52
Total major complications	23 (48.93%)	11 (22%)	0.005
Revision surgery (THR and hardware removals)	13 (27.65%)	9 (18%)	0.25
THR out of revision surgery	13 (27.65%)	8 (16%)	0.16

Categorial variables are presented as count (percent).

**Table 3:** Quality-of-life measurements. The Brunner–Munzel test was used to test for statistical significance between the groups.

	Pinning Procedure (*n* = 47)	Targon FN (*n* = 50)	*p* Value
Chronic pain—*n* (%)			
No pain	3 (6.38%)	7 (14%)	0.21
Mild pain	9 (19.14%)	16 (32%)	
Moderate pain	2 (4.25%)	7 (14%)	
Severe pain	1 (2.12%)	5 (10%)	
Extreme pain	0 (0%)	3 (6%)	
Ambulation ability—*n* (%)			
No problem	7 (14.89%)	30 (60%)	0.07
Slight problem	4 (8.51%)	5 (10%)	
Moderate problem	2 (4.25%)	1 (2%)	
Severe problem	0 (0%)	1 (2%)	
No ability to walk	1 (2.12%)	1 (2%)	
Ambulation assistive device—*n* (%)			
No walking aids	7 (14.89%)	29 (58%)	0.07
One aid	3 (6.38%)	8 (16%)	
Two aids	1 (2.12%)	0 (0%)	
Walking frame	1 (2.12%)	0 (0%)	
Wheelchair	2 (4.25%)	1 (2%)	

Ordinal variables are presented as count (percent).

## Data Availability

The data presented in this study are available on request from the corresponding author.
